# Smoking-Attributable Direct Healthcare Expenditure in Lithuania: A Prevalence-Based Annual Cost Approach

**DOI:** 10.3390/medicina54020015

**Published:** 2018-04-12

**Authors:** Vaida Liutkutė, Mindaugas Štelemėkas, Aurelijus Veryga

**Affiliations:** 1 Health Research Institute, Faculty of Public Health, Medical Academy, Lithuanian University of Health Sciences, Tilžės g. 18, LT-47181 Kaunas, Lithuania; mindaugas.stelemekas@lsmuni.lt; 2 Ministry of Health of The Republic of Lithuania, Vilniaus g. 33, LT-01506 Vilnius, Lithuania; aurelijus.veryga@gmail.com

**Keywords:** smoking, healthcare, expenditure, economic cost

## Abstract

*Introduction*: The estimates of the economic burden of smoking provide the basis for a comprehensive assessment of the overall economic impact and evidence for potential public health policy intervention by the government. The aim of this paper is to estimate the smoking-attributable direct healthcare expenditure covered by the Compulsory Health Insurance Fund (CHIF) in Lithuania in 2013. *Methods*: A prevalence-based and disease-specific annual cost approach was applied to 25 smoking-related diseases or disease categories. Our analysis included only direct government healthcare expenditure (reimbursed by CHIF), including: smoking-attributable outpatient and inpatient care services, medical rehabilitation, reimbursable and publicly procured pharmaceuticals and medical aids, the emergency medical aid (ambulance) service, nursing, and expensive tests and procedures. The smoking-attributable expenditure on the above-mentioned healthcare services was calculated by multiplying the total annual expenditure by the corresponding smoking attributable fractions (SAFs). *Results*: The total smoking-attributable government expenditure amounted to €37.4 million in 2013. This represented 3% of the total CHIF budget in 2013. Smoking-attributable expenditure on inpatient care and medical rehabilitation services was two times higher for male smokers, than for female smokers. *Conclusions*: Smoking imposes a significant preventable financial cost within the budget of the Lithuanian healthcare system. A quantitative estimation of smoking related healthcare costs could provide an incentive for the development of smoking cessation services, with additional attention towards male smokers, as well as an important focus on smoking prevention among children and youths.

## 1. Introduction

In addition to damaging health, the use of tobacco imposes costs, not only on individuals, but also on society as a whole; therefore, an accurate assessment of smoking-attributable economic costs is essential for implementing evidence-based governmental public health policy interventions. The World Health Organization (WHO) identifies the assessment of the cost of smoking as a high research priority [1]. Smoking-attributable healthcare spending is an important component of smoking-attributable overall economic cost.

It is estimated that the global economic costs of smoking-attributable diseases (from both health expenditures and productivity losses) were US$ 1436 billion in 2012, equivalent in magnitude to 1.8% of the world’s annual gross domestic product (GDP), whereas in Eastern Europe the economic burden of smoking amounts to 3.6% of GDP, compared to 2% in the rest of the Europe and 0.6% in the Eastern Mediterranean. In terms of health expenditure, both the Americas and Europe have the heaviest burden, while in Eastern Europe, where the tobacco epidemic is generally the most severe, smoking might be responsible for around 10% of the total healthcare expenditure (THE) [2].

Like many other European countries, Lithuania has a national compulsory health insurance system, stipulating that residents of Lithuania are obliged to pay health insurance contributions; about 60% of population were fully insured by the state in 2013. Since 1997, the National Health Insurance Fund under the Ministry of Health (NHIF) has been the main agent responsible for financing the health system. The Ministry of Health determines which services are covered by the NHIF, based on the Health Insurance Law, and these payment mechanisms establish the rules for provider contracts, budgeting, and financial management decisions; they also set the rules of healthcare provision and, reference prices (in points) for the reimbursement of healthcare services and pharmaceuticals [3].

Lithuania had a total resident population of three million in 2013. According to Statistics Lithuania, daily smoking among men aged 15 and older decreased from 42% in 2005 to 34% in 2014, and among women declined from 10% to 9% accordingly [4,5]. Furthermore, the latest round of the Health Behaviour in School-aged Children study has demonstrated that in Lithuania the number of 15-year-olds who smoke at least once a week decreased by 14% among boys and 9% among girls [6,7]. Despite some positive trends in prevalence, smoking remains one of the major behavioral risk factors causing a substantial number of potentially preventable deaths. For instance, active smoking alone caused one out of seven deaths in Lithuania in 2013 [8].

The main objective of this study was to estimate the active smoking-attributable direct healthcare expenditure paid by the CHIF in Lithuania in 2013. To our knowledge, this is the first national attempt to quantify healthcare expenditure associated with the treatment of smoking-related diseases.

## 2. Experimental Section

This study follows the classic cost-of-illness approach as applied by Rice et al. [9]; this defines economic costs as either a direct or an indirect costs. The latter is not a subject of this study. This analysis only targets the direct government expenditure on personal healthcare services including smoking-attributable outpatient and inpatient care services, medical rehabilitation, reimbursable and publicly procured pharmaceuticals, medical aids, the emergency medical aid (ambulance) service, nursing, and expensive tests and procedures.

The annual cost approach of prevalence-based disease determines the proportion of excess cost that can be attributed to tobacco use and hence be attributed as preventable. We applied the prevalence-based approach and the concept of smoking attributable fraction (SAF) to the 25 diseases or disease categories (see Table 1) that have a significant association between smoking and the risk of mortality. Smoking-related diseases and disease-specific relative risks (RRs) were obtained from the analyses of the Cancer Prevention Study II (CPS-II) and the updated analyses of the pooled contemporary cohort population that was published in the 2014 US Surgeon General’s report [10]. Because the health effects of smoking result from many years of exposure, most studies evaluating the burden of smoking focus on adults aged 35 and older. We also followed this approach and calculated SAFs only for the age groups of 35–54; 55–64; 65–74; ≥75. The relative mortality risks of cigarette smoking were used as a proxy for the relative morbidity risks of cigarette smoking resulting from a lack of data. Gender- and age group-specific prevalence rates of current, former, and non-smokers were obtained from the 2005 Health Interview Survey carried out by Statistics Lithuania. We thereby applied an eight-year lag to our calculations of the SAF. A more detailed description of the methodology of calculating SAFs for Lithuania, as well as methodological challenges, is published elsewhere [8], whereas gender- and disease-specific SAFs are provided in Table 1.

At the planning stage of the research, data from 2013 was the latest available.

### 2.1. Expenditure on Outpatient Care Services

In our analysis, smoking-related expenditure on outpatient care includes: (1) delivered primary outpatient healthcare services and (2) outpatient services (such as outpatient specialist consultations, outpatient services provided in hospital emergency departments, day care, day surgery, observation, and outpatient surgery). The disease-specific expenditure on the latter services (in thousands of points) was obtained from the NHIF. Age- and gender-specific data was not available. The reference prices paid from the budget of the CHIF were approved in points; the value of the points for different healthcare services was approved by the Minister of Health of the Republic of Lithuania. In 2013, the value of one point was equal to 0.89 LTL which is equal to €0.26 per one point for outpatient consultations, inpatient healthcare services, medical rehabilitation, and sanatorium treatment [11]. The smoking-attributable expenditure on provided outpatient services was calculated by multiplying the total annual disease-specific cost in the overall population by the correspondent disease-specific SAFs. Meanwhile, primary outpatient healthcare expenditure in Lithuania is paid according to the factual number of patients registered with the healthcare provider. According to the NHIF, the expenditure on primary outpatient healthcare services (except primary outpatient dental healthcare and primary outpatient mental healthcare) amounted to €170.4 million in 2013. Neither age and gender, nor disease-specific data on primary outpatient care services was available. Thus, we presumed that the proportion of smoking-attributable expenditure on primary outpatient healthcare services was equal to the estimated proportion of the smoking-attributable provided outpatient services expenditure.

### 2.2. Expenditure on Inpatient Care Services and Medical Rehabilitation

Smoking-related expenditure on inpatient care includes: (1) long-term treatment (inpatient nursing and supportive treatment services) and (2) inpatient active treatment services. Meanwhile, medical rehabilitation includes: (1) inpatient and (2) outpatient rehabilitation services.

Gender, age (35–54; 55–64; 65–74; ≥75), and smoking-related disease-specific hospitalization days were calculated using primary data from the CHIF information system “SVEIDRA”. Firstly, considering the number of derived hospitalization days and using reference prices, approved in points [12,13,14], we calculated the total expenditure in points. Then, after applying the above-mentioned (Section 2.1) one point value, we estimated smoking-attributable expenditure on inpatient treatment multiplying them by the correspondent gender, age, and disease-specific SAFs. It is important to mention that in 2012 Lithuania begun to gradually implement the method of diagnosis-related groups (DRGs) for the reimbursement of active inpatient treatment services. Thus, we calculated the price of active treatment hospitalization days in points using the DRG method [15]. Both types of inpatient care services, as well as inpatient and outpatient medical rehabilitation, were analyzed separately.

### 2.3. Expenditure on Pharmaceuticals and Medical Aids

The funding for pharmaceuticals from the CHIF budget consists of the costs of the reimbursable pharmaceuticals, publicly procured pharmaceuticals, and medical aids. Following the same pattern as in the previous estimation, we applied corresponding SAFs to the total disease-specific expenditure (in Euros) for reimbursable pharmaceuticals and medical aids. Unfortunately, the electronic record system of public procurement of pharmaceuticals and medical aids was set up in 2013 and disease-specific data for these types of pharmaceuticals was not available. Hence, we once again made the presumption that the proportion of smoking-attributable expenditure on public procurement of pharmaceuticals and medical aids was equal to the estimated proportion of the smoking-attributable reimbursable pharmaceuticals and medical aids expenditure.

### 2.4. Other Expenditure

Finally, we included governmental expenditures for the emergency medical aid (ambulance) service, nursing services (such as nursing and supportive treatment, palliative aid, services of nursing at home, and services of nursing people with diabetes mellitus), and expensive tests and procedures to our analysis. It should further be noted that expensive tests and procedures according to the NHIF are limited to such medical procedures as: computerized tomography, magnetic resonance tomography, hyperbaric oxygen chamber, gravitational surgery of the blood, hemodialysis, immunotyping, genetic testing, and coagulation factors analyses [16]. We obtained the annual expenditure on the latter services from the NHIF. Consequently, we presumed that the estimated proportion of the smoking-attributable expenditure on the medical rehabilitation services was equal to the proportion of smoking-attributable expenditure on the emergency medical aid (ambulance) service, nursing services, and expensive tests and procedures. This methodological approach is discussed in the discussion section in more detail.

### 2.5. Data Sources

A detailed expenditure of the CHIF 2013 budget was provided by the NHIF. The data of the resident population at the beginning of the year of 2013, according to age groups and gender, were obtained from Statistics Lithuania [17]. We used the official conversion rate (3.45280 LTL per €1) as defined by the Council of the European Union [18].

## 3. Results

Table 2 shows the smoking-attributable direct healthcare expenditure, according to groups of smoking-related diseases, included in our estimation.

The total active smoking-attributable direct healthcare expenditure, covered by the government, reached €37.4 million in 2013 (see Table 2), with more than half of these costs (67%) given for inpatient hospitalizations. The estimated smoking-attributable expenditure represented 3% of the total €1.2 billion CHIF budget in 2013 [19] or approximately 0.1% of Lithuania’s GDP of the same year [20]. For illustrative purposes, the smoking-attributable expenditure estimates, in comparison to the total CHIF budget expenditure, are presented in Table 3.

The smoking-attributable expenditure on inpatient care and medical rehabilitation services among male smokers (aged 35 and older) was two times higher compared to female smokers (€18,036.6 and €7,544.4 respectively). Expenditure on Ischemic Heart Disease was highest (for both male and female) and amounted to 39% of all smoking-attributable expenditures on these services.

## 4. Discussion

Direct healthcare costs, due to smoking, amounted to at least €37.4 million in Lithuania in 2013. This represented 3% of the total annual governmental expenditure on healthcare services, reimbursed by the CHIF.

Although the negative health consequences of smoking are already known, the evidence for the economic consequences is still relatively unknown. The existing estimates of the smoking-attributed social and economic burden are fragmented. It is estimated that active smoking caused 5771 deaths, and determined the loss of over 39 thousand years of potential life [8] in Lithuania in 2013. It is also estimated that productivity loss, due to premature mortality, is around €12 million annually [21].

Our estimated smoking-attributable healthcare costs are very likely to be underestimated for several reasons. Firstly, for the purpose of this study, we limited our estimation to active smoking only. The Global Burden of Disease study identified the trend that the proportion of secondhand smoke-attributable deaths and disability-adjusted life-years in Eastern Europe is the highest in the European region [22]. Thus, inclusion of secondhand smoking would very likely additionally increase the costs.

Secondly, this analysis only assessed the state budget expenditure, not taking into account the private expenditure for self-treatment, out-of-pocket payments, co-payments, and private insurance. The review of the Lithuanian healthcare system in 2013 identified that the out-of-pocket expenditure constitutes 26% of the total expenditure on health, more than 70% of which is for pharmaceuticals [3]. The comparison of the health expenditure by the payment source could have resulted in a more accurate overall estimation of the smoking-attributable direct healthcare expenditure.

Thirdly, it is possible that some of the methodological choices we made could have easily meant that we underestimated the smoking-attributable expenditure. For example, we estimated the expenditure of the emergency medical aid (ambulance) service, nursing services, and expensive tests and procedures by applying a proportion of the smoking-attributable medical rehabilitation. Because of the gender, age, and disease-specific data availability of medical rehabilitation and inpatient care hospitalization days, estimations of these smoking-attributable expenditures may be considered as mostly accurate. The proportion of the smoking-attributable expenditure on inpatient care was equal to 5.9% of the total expenditure on these services and the estimated proportion of smoking-attributable expenditure on medical rehabilitation was 1.7%. We chose to use a smaller proportion (1.7%) to keep a reasonable approximate conservative estimation. Smoking-attributable expenditure on the above-mentioned services could have reached €6.7 million (3.5 times more) if we had used the higher proportion (5.9%). This could have increased the overall proportion of the total smoking-attributable expenditure by 0.3%. The somewhat methodologically arbitrary decision to apply proportions has its shortcomings, but herewith allowed us to cover the vast majority of the expenditures paid by the CHIF and include more smoking-related healthcare system costs into the current estimation.

Because of the non-availability of some data required for the analysis, this study is subject to some more limitations. We used the RR of death as a proxy to estimate the direct healthcare cost of smoking. This approach has also been used in other cost studies [23,24,25,26,27,28,29,30,31], as well as being listed in the WHO toolkit: “Economics of tobacco toolkit: assessment of the economic costs of smoking” [32]. Furthermore, we used RRs based on data from studies carried out in other countries. The CPS-II is one of the largest smoking and mortality studies ever conducted. It provides separate RR estimates for different causes of death, and most smokers studied were lifelong cigarette smokers, which allowed the full effects of the smoking epidemic to be captured.

The limitations of using US-based RRs have been noted by other authors [33,34] and applied to our study as well. Furthermore, using a population-based attributable fraction, we assume that the proportion of persons exposed to smoking among those admitted to hospitals is the same as the proportion in the general population. According to some authors [35], such an assumption is likely to result in an underestimation of the true healthcare costs, since smokers are usually over-represented among healthcare clients.

One of the main strengths of this study is the use of the updated list of smoking-related diseases and RRs published in the 2014 US Surgeon General’s Report. This report showed that now there is sufficient evidence to infer a causal relationship between smoking and five additional diseases in adults: age-related macular degeneration, diabetes mellitus, tuberculosis, liver cancer, and colorectal cancer [10]. All of these diseases, except age related macular degeneration, were included in our estimation, making a total of 25 diseases or disease categories.

Despite the limitations and the above-mentioned weaknesses, which we believe result in an underestimation of the smoking-attributable costs in Lithuania, we established that active smoking alone imposes a significant financial burden on Lithuanian society. It is important to note that even the conservative estimate presented by this analysis is huge enough in comparison to the taxes collected from tobacco. In 2013, nearly €212 million were collected in excise taxes for manufactured tobacco [36], and only €0.323 million were collected as charges and fees for the licenses for the retail of tobacco products [37]. Considering that our estimated financial burden (€37.4 million) includes only one component of smoking-attributed economic burden, the actual financial burden may easily exceed the income to the state from this economic activity, especially if the secondhand smoking data are included in the analysis.

The share of THE spent on treatment of smoking-related diseases in Lithuania is comparable to the estimate (3.1% THE and 0.2% GDP, in 2009) reported in “A study on liability and the health costs of smoking” [38], but lower (8.3% THE and 3.2% GDP, in 2012) compared to that reported by Goodchild et al. [2]. Although comparison of such findings requires a detailed analysis of the applied methods, there is at least one reason to explain why the estimates for 2012 and 2013 are so different. The Goodchild et al. [2] study applied the proportion of smoking-attributable healthcare expenditure as estimated for 2000 [39] to Lithuania’s THE in 2012; the higher SAFs in 2000 because of a higher prevalence of smoking could be the main reason for such a significant difference.

Lightwood et al. concluded that changes in healthcare expenditure appear soon after changes in smoking behavior [40]. Therefore, estimates of the direct smoking-attributable healthcare costs help to understand the economic impact of smoking, and should motivate policymakers to implement evidence-based comprehensive tobacco control policies that are proven to reduce the health and economic burden. Key evidence-based tobacco control and prevention interventions, also known as policy “best buys” (including tobacco product price increases; comprehensive smoke-free laws; warnings and health information about the effects of tobacco; enforcement of bans on tobacco advertising, promotion and sponsorship), could prevent smoking initiation, reduce cigarette consumption and increase the number of successful quitters [41,42,43,44,45].

A recent study conducted in Lithuania has shown that the progress in the Lithuanian tobacco control policies since 2000 was associated with an increase in smoking cessation, benefitting both highly educated and lower educated groups [46]. Furthermore, our analysis clearly points at a significantly higher (two times) expenditure for inpatient care and medical rehabilitation in male as opposed to female smokers, which supports previous national findings related to smoking-attributable mortality. It was estimated, that in 2013 smoking caused one out of 35 deaths among females and one out of four among males [8]. Such findings oblige the state to improve cessation services for smokers with additional efforts focused towards male smokers. Gender specific and overall interventions, such as price increases for tobacco products, are well known as evidence based measures when aiming to reduce the burden of smoking on society and complies well with the implementation of The Framework Convention on Tobacco Control on a country level.

As already mentioned, the healthcare costs of smoking distinguish between direct and indirect costs, based on the prevalence-based cost-of-illness approach. Indirect costs represent the economic loss due to the morbidity caused by smoking-related diseases and usually involve a calculation of the present value of the loss of labor productivity. This study was limited to direct costs only, thus a comprehensive national estimation of the indirect healthcare costs and most importantly the overall economic and social burden of smoking remains lacking. Further research should include the secondhand smoking-attributable costs, including those of children exposed to their parents’ smoking. Another no less important challenge for future research is the inclusion of Electronic Nicotine Delivery Systems and Electronic Non-Nicotine Delivery Systems (such as e-cigarettes and heat-not-burn tobacco products). The use of e-cigarettes is increasing among various age groups across the globe [47,48,49], and therefore, it is becoming an epidemiological challenge to the whole research community. As a new worldwide health risk, e-cigarettes will sooner or later become a new research object for economic burden studies.

## 5. Conclusions

Smoking imposes a significant financial loss in the Lithuanian healthcare system’s budget. Up to date quantitative estimations provide evidence for the need of interventions on the behalf of public and incentives for developing smoking cessation services, with additional attention focused towards male smokers, as well as an important focus on smoking prevention among children and youths. Hopefully, this study can be considered a starting point and will provide impetus for a comprehensive assessment of the social and economic burden of smoking exposure in Lithuania.

## Figures and Tables

**Table 1:** Smoking-attributable fraction (% SAF) for both current and former smokers aged 35 and older according to sex and smoking-related disease in Lithuania in 2013.

Disease Category	ICD-10 Code	% SAF		
		Male	Female	Both
**Cancer**				
Lip, Oral Cavity, Pharynx	C00–C14	29.4	6.2	16.1
Esophagus	C15	29.4	6.2	16.1
Stomach	C16	29.4	6.2	16.1
Pancreas	C25	29.4	6.2	16.1
Larynx	C32	29.4	6.2	16.1
Trachea, Lung, Bronchus	C33–C34	85.4	56.1	68.6
Cervix Uteri	C53	0.0	6.2	3.6
Kidney and Renal Pelvis	C64–C65	29.4	6.2	16.1
Urinary Bladder	C67	29.4	6.2	16.1
Acute Myeloid Leukemia	C92.0	29.4	6.2	16.1
Colorectal	C18–C20	29.4	6.2	16.1
Liver	C22	29.4	6.2	16.1
**Cardiovascular and Cerebrovascular Diseases**				
Ischemic Heart Disease	I20–I25	46.8	26.6	35.2
Acute Rheumatic Fever and Rheumatic Heart Disease	I00–I09	7.7	0.7	3.7
Other Heart Disease	I26–I51	7.7	0.7	3.7
Cerebrovascular Disease	I60–I69	6.8	0.6	3.3
Atherosclerosis	I70	17.2	3.2	9.2
Aortic Aneurysm	I71	17.2	3.2	9.2
Other Arterial Disease	I72–I78	17.2	3.2	9.2
**Respiratory Diseases**				
Pneumonia, Influenza	J10–J18	49.3	34.1	40.6
Bronchitis	J40–J42	49.3	34.1	40.6
Emphysema	J43	49.3	34.1	40.6
Chronic Airway Obstruction	J44	49.3	34.1	40.6
**Other**				
Diabetes Mellitus	E10–E14	3.5	0.3	1.7
Tuberculosis	A16–A19	49.3	34.1	40.6

**Table 2:** Smoking-attributable direct healthcare expenditure by disease category and healthcare service type in Lithuania in 2013 (EUR).

	Cancer ^a^	Cardiovascular and Cerebrovascular Diseases ^b^	Respiratory Diseases ^c^	Other ^d^	All Cause
Inpatient * care services					
-long-term treatment	369,923	1,251,146	41,918	210,136	1,873,123
-inpatient active treatment	4,063,324	11,561,888	7,321,412	78,058	23,024,683
**Outpatient care services**					
-primary outpatient healthcare					511,172
-provided outpatient service	177,522	213,054	168,482	21,426	580,484
**Medical rehabilitation**					
-inpatient rehabilitation	82,898	578,959	9064	4409	675,330
-outpatient rehabilitation	712	4937	2240	7	7895
**Pharmaceuticals**					
-reimbursable pharmaceuticals and medical aids	2,615,651	1,937,661	2,230,334	434,300	7,217,946
-publicly procured pharmaceuticals and medical aids					1,353,594
**Other**					
-emergency medical aid (ambulance) service					689,040
-nursing					703,270
-expensive tests and procedures					535,560
**Total**	7,310,030	15,547,645	9,773,449	748,337	37,354,125

The numbers in the table are rounded and so may not correspond to totals. * Healthcare expenditures on inpatient care services and medical rehabilitation were calculated for males and females aged ≥35. Meanwhile, the rest of the calculations are based on the overall population data. ^a^ Cancer (ICD-10 codes: C00–C14; C15; C16; C25; C32; C33-C34; C53; C64–C65; C67; C92.0; C18-C20; C22). ^b^ Cardiovascular and Cerebrovascular diseases (ICD-10 codes: I20–I25; I00–I09; I26–I51; I60–I69; I70; I71; I72–I78). ^c^ Respiratory diseases (ICD-10 codes: J10–J18; J40–J42; J43; J44). ^d^ Other (ICD-10 codes: E10–E14; A16–A19).

**Table 3:** The proportion of the Compulsory Health Insurance Fund expenditure attributable to active smoking in Lithuania in 2013.

	SAHE (€)	THE (€)	%THE
Inpatient * care services	24,897,806	424,056,045	5.9
Outpatient care services	1,091,684	336,437,791	0.3
Medical Rehabilitation	683,226	39,243,513	1.7
Pharmaceuticals and medical aids	8,753,540	235,547,961	3.7
Emergency medical aid (ambulance) service	689,040	40,531,782	1.7
Nursing	703,270	41,368,797	1.7
Expensive tests and procedures	535,560	31,503,545	1.7
Total	37,354,125	1,148,689,433	3.3 **

SAHE—Smoking-attributable health expenditure; THE—Total healthcare expenditure. The numbers in the table are rounded and so may not correspond to totals. * Healthcare expenditure on inpatient care services and medical rehabilitation were calculated for males and females aged ≥35. The rest of calculations were based on the overall population data. ** These numbers reflect only estimated total healthcare expenditure for the healthcare services included in the analysis and listed in Table 2. The total Compulsory Health Insurance Fund (CHIF) budget in 2013 was €1,232.7 million and %THE was 3%.
